# Eucalyptus Globulus in Intermittent Fever

**Published:** 1873-04

**Authors:** 


### EUCALYPTUS GLOBULUS IN INTERMITTENT FEVER.

About two years ago, Dr. Lorinser, of Vienna, laid before the profession the results of his observations on the treatment of ague by the *Eucalyptus Globulus* (see *British Medical Journal*, May 21, 1870). A supply of the tincture was placed, for the purpose of observation, at the disposal of medical men connected with the railway stations in localities where ague was frequent. The quantities, however, were but small, and a larger supply was delivered in May of last year. The results obtained during the summer have been collected and summarized by Dr. Joseph Keller, Chief Physician of the Austrian Railway Company.

The number of patients treated with tincture of eucalyptus was 342. Of these, 310 (71.76 per cent.) were perfectly cured, and 122 (28.24) required to be afterwards treated with quinine. Of the 310 patients who were cured, no paroxysm occurred after the first dose in 202; in the remaining 108, there were one or more subsequent paroxysms, which, however, yielded to repeated doses of the medicine. Quinine had been given, without result, in 118 of the 432 cases; 293 of the patients had had ague in previous years, and 139 were attacked for the first time in 1871. Of the 122 cases in which eucalyptus failed, 58 recovered under the use of quinine, 38 were not cured, 10 were sent home, and 16 remained under treatment. Of the 118 cases in which quinine had been given unsuccessfully, 91 recovered under the use of eucalyptus, and in 27 no result followed.

The several types of intermittent fever were represented as follows: Quotidian, complicated, 117—simple, 73=190; tertian, complicated, 126—simple, 95=221; quartan, complicated, 16—simple, 4=20; quintan, complicated, 1. The complications were enlargement of the spleen or liver, anæmia or chronic gastric catarrh, paludal cachexia, etc. The remedy was successful in 161 of the 260 complicated cases, or 61.9 per cent.; and in 149 (or 86.6 per cent.) of the 172 simple cases. The percentage of success in the several types were: In tertian, 75.57; in quartan, 70; in quotidian, 67.89. Among the cases in which the first dose of eucalyptus arrested the disease, were 95 complicated and 107 simple; 28 of the former and 20 of the latter had previously been treated unsuccessfully with quinine. In the cases where the paroxysms recurred, there were 70 complicated and 38 simple; quinine had been given without success in 27 of the former and in 15 of the latter.

Of the 432 patients, 353 were men, 46 women, and 33 children. There were 155 patients who were immigrants into the localities, and in these the disease was more frequently attended with complications, and the treatment was less successful, than among the indigenous inhabitants.

The treatment was generally commenced on the fifth day after the first paroxysm of ague; its duration averages 9½ days, that with quinine in previous years having been 12½ days.

The tincture was made by dividing into small pieces the leaves of eucalyptus obtained through France from the native country of the plant, and macerating in alcohol for three months. Ten pounds of leaves yielded twenty-five quarts of the tincture. The average dose was two drachms, and the average quantity used for each patient was seven drachms; this, however, varied much, according to the nature of the case and its complications.

Dr. Keller concludes that eucalyptus must be regarded as a very important remedy for ague, but that the plant as cultivated in Austria is less efficacious than that imported from the native soil; that the remedy is of service especially in obstinate cases of ague, where quinine has been given unsuccessfully; and that the average duration of treatment by eucalyptus is shorter than that by quinine. He believes that the tincture is the most eligible preparation of the plant, as the essential oil is retained. The cost of a quart of the tincture he calculates to be less than two florins. It has a pleasant aromatic flavor. For women and children, some simple or orange syrup may be added. In milder cases, two or three teaspoonsful, taken before the expected paroxysm, are generally sufficient. Where cachexia is present, small doses should be taken night and morning for some time.—*Mich. University Med. Jour.*

